# Robust error calibration for serial crystallography

**DOI:** 10.1063/4.0000907

**Published:** 2025-10-27

**Authors:** David W. Mittan-Moreau, Vanesa Oklejas, Daniel W. Paley, Asmit Bhowmick, Jan Kern, Nicholas K. Sauter, Aaron S. Brewster

**Affiliations:** Lawrence Berkeley National Lab, Berkeley, CA, USA

## Abstract

Serial crystallography is an important technique with unique abilities to resolve enzymatic transition states, minimize radiation damage to sensitive metalloenzymes, and perform de novo structure determination from micron sized crystals. This technique requires the merging of data from thousands of crystals, making manual identification of errant crystals infeasible. cctbx.xfel.merge uses filtering to remove problematic data. However, this process is imperfect, and data reduction must be robust to outliers. We add robustness to cctbx.xfel.merge at the step of uncertainty determination for reflection intensities. This step is a critical point for robustness because it is the first step where datasets are considered as a whole, as opposed to individual lattices. Robustness is conferred by reformulating the error calibration procedure to have fewer and less stringent statistical assumptions and incorporating the ability to down weight low quality lattices. We then apply this method to five macromolecular XFEL datasets and observe improvements to each. The appropriateness of the intensity uncertainties is demonstrated through internal consistency. This is performed through theoretical CC ½ and I/σ relationships and by weighted second moments, which use Wilson's prior to connect intensity uncertainties with their expected distribution. This work presents new mathematical tools to analyze intensity statistics and demonstrates their effectiveness through the often underappreciated process of uncertainty analysis.

